# Synthesis and structure of tetra­aqua­bis­(dimethyl ether)magnesium(II) dibromide dimethyl ether disolvate

**DOI:** 10.1107/S205698902600366X

**Published:** 2026-04-14

**Authors:** Beata Moritz, Tristan Mairath, Carsten Strohmann

**Affiliations:** a TU Dortmund University, Department of Chemistry and Chemical Biology, Inorganic Chemistry, Otto-Hahn-Strasse 6, 44227 Dortmund, Germany; University of Aberdeen, United Kingdom

**Keywords:** crystal structure, magnesium(II) bromide, dimethyl ether, Hirshfeld surface analysis, hydrogen bonds

## Abstract

Unlike typical hexa­hydrates, the title compound, [Mg(C_2_H_6_O)_2_(H_2_O)_4_]Br_2_·2C_2_H_6_O or [Mg(H_2_O)_4_(DME)_2_]Br_2_·DME_2_ (DME = dimethyl ether, C_2_H_6_O), is a water-poor magnesium(II) complex. The central magnesium cation (site symmetry 1) is coordinated by four water mol­ecules and two mol­ecules of dimethyl ether and adopts a slightly elongated *trans*-octa­hedral coordination geometry. The water mol­ecules are linked to outer-sphere bromide anions and additional dimethyl ether mol­ecules *via* O—H⋯Br and O—H⋯O hydrogen bonds. Due to the volatility of dimethyl ether, the presence of coordinating and non-coordinating mol­ecules of this ether makes this solid state structure presented here particularly inter­esting. To investigate the inter­molecular inter­actions leading to this special coordination, a Hirshfeld surface analysis was performed. It showed that the H⋯H inter­actions (70.4%) make the largest contribution to the crystal packing, followed by H⋯Br inter­actions (19.4%), H⋯O inter­actions (10.1%) and Br⋯O inter­actions (0.1%).

## Abstract 2

Unlike typical hexa­hydrates, the title compound, [Mg(C_2_H_6_O)_2_(H_2_O)_4_]Br_2_·2C_2_H_6_O or [Mg(H_2_O)_4_(DME)_2_]Br_2_·DME_2_ (DME = dimethyl ether, C_2_H_6_O), is a water-poor magnesium(II) complex. The central magnesium cation (site symmetry 1) is coordinated by four water mol­ecules and two mol­ecules of dimethyl ether and adopts a slightly elongated *trans*-octa­hedral coordination geometry. The water mol­ecules are linked to outer-sphere bromide anions and additional dimethyl ether mol­ecules *via* O—H⋯Br and O—H⋯O hydrogen bonds. Due to the volatility of dimethyl ether, the presence of coordinating and non-coordinating mol­ecules of this ether makes this solid state structure presented here particularly inter­esting.

## 1. Chemical context

Magnesium(II) bromide is a well-known chemical with a wide range of applications. For example, it can be used to catalyze nucleophilic addition reactions as a Lewis acid in organic synthesis (Annunziata *et al.*, 1992 ▸). It is also reported to catalyze cyclo­additions (Danishefsky *et al.*, 1985 ▸) and rearrangement reactions (Black *et al.*, 1988 ▸, 1990 ▸). Furthermore, magnesium(II) bromide is known for its use in polymerization reactions (Daito *et al.*, 2021 ▸) or possible catalytic effect on the formation of Grignard reagents (Garst *et al.*, 1994 ▸).

While magnesium(II) bromide is a commonly used salt, and many complexations of MgBr_2_-containing compounds with etheric solvents like THF are known (Seyferth, 2009 ▸; Toney & Stucky, 1971 ▸), the solvent considered here, dimethyl ether (C_2_H_6_O; DME), exhibits challenging properties. DME, with a boiling point of 248 K (Bauer & Kruse, 2019 ▸), is the smallest ether available. Nevertheless, it can be used, for example, as an extraction solvent (Bauer & Kruse, 2019 ▸; Zheng & Watanabe, 2022 ▸) or as an alternative to conventional fuels (Semelsberger *et al.*, 2006 ▸; Catizzone *et al.*, 2021 ▸). However, with regard to chemical synthesis and structural studies, it has been less investigated.

This is consistent with the absence of solid-state structures involving dimethyl ether, and is particularly evident from the fact that only one other solid-state structure of a magnesium(II) complex with dimethyl ether as a ligand (**2**) is known to date. In this work, the title compound (**1**), which represents the second structure of a magnesium(II) complex containing dimethyl ether is reported. In complex **2**, the magnesium cation is coordinated by two dimethyl ether mol­ecules and two bidentate B_3_H**_8_**^−^ ligands (CSD refcode KIRWAK; Kim *et al.*, 2007 ▸), resulting in a distorted MgO_2_H_4_*cis*-octa­hedral geometry. The magnesium center in complex **1** adopts a *trans*-octa­hedral geometry. Compared to the magnesium complexes with dimethyl ether as ligands, which have been less studied to date, the structural motif of magnesium(II) hexa­hydrates like **3** is well known (*e.g*., YIKLAH; Hennings *et al.*, 2013 ▸). Such structures can be described as water-rich, whereas compound **1** represents a relatively water-poor compound. Complex **1** is described in more detail below, providing an overview of its structure and crystal packing.
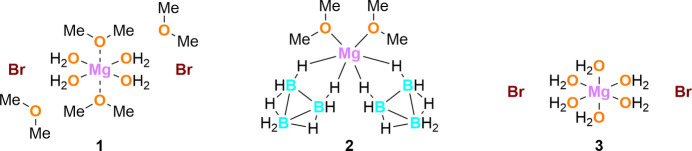


## 2. Structural commentary

**Figure 1:**
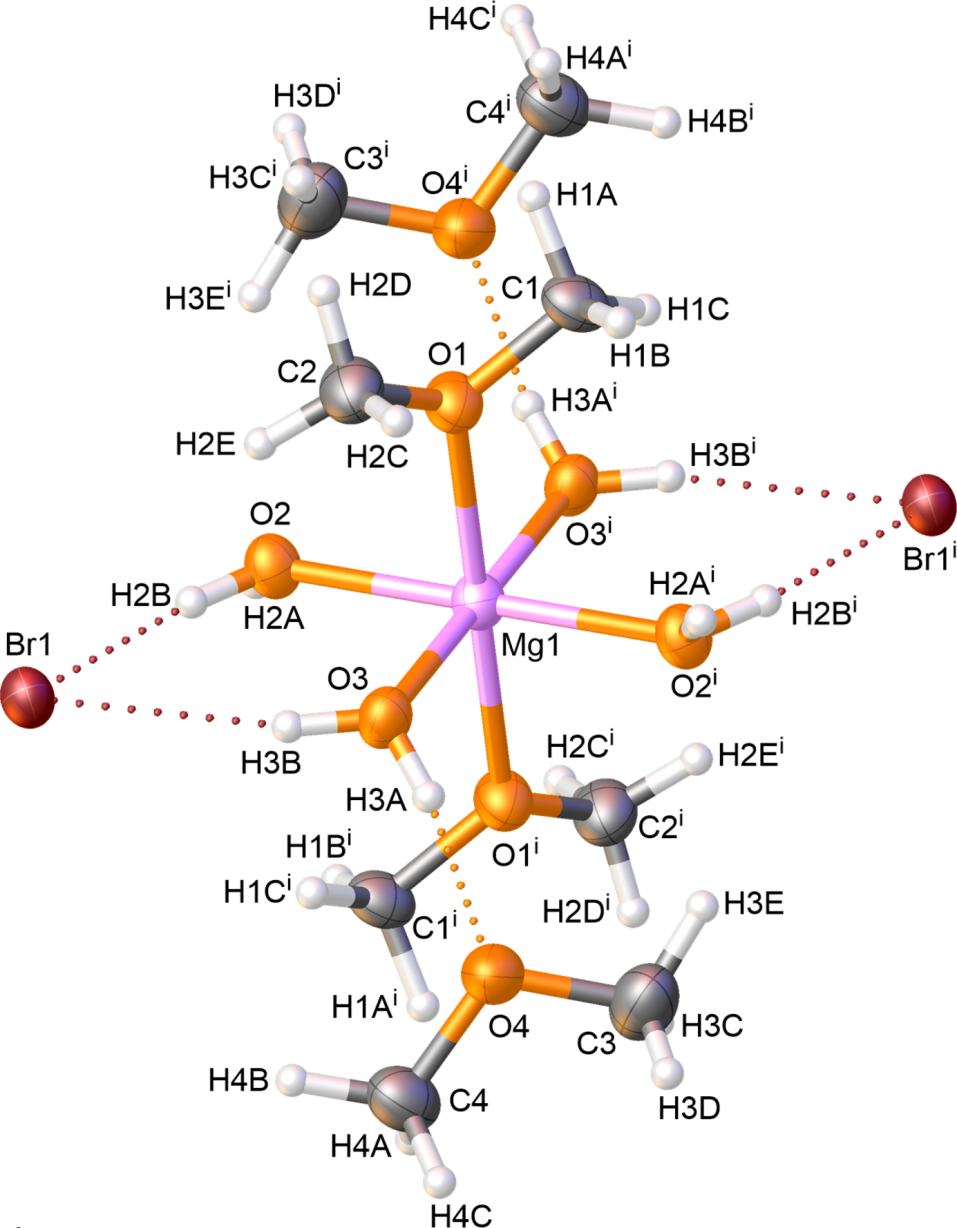
The mol­ecular structure of **1**, showing the atom labeling and 50% probability displacement ellipsoids. Symmetry code: (i) −*x* + 1, 1−*z* + 1, −*z* + 1.

Complex **1**, [Mg(H_2_O)_4_(DME)_2_]Br_2_·DME_2_, crystallizes at 193 K in the monoclinic space group *P*2_1_/*n* (Fig. 1 ▸). The asymmetric unit consists of one half of the complex with the magnesium cation lying on the inversion center at 1/2, 1/2, 1/2 for the asymmetric atoms and the second half is generated by inversion symmetry. The metal ion in **1** exhibits a slightly distorted MgO_6_ octa­hedral coordination geometry with two bromide anions and two dimethyl ether mol­ecules located in the outer sphere. This geometry can be identified by the angles around the magnesium center, which are close to 90° (Table 1 ▸). In this arrangement, the water mol­ecules are in the equatorial plane. The distances between the water oxygen atoms (O2 and O3) and the metal center are very similar to each other. In contrast, the distances between the magnesium atom and the directly coordinating (*via* O1), axially positioned dimethyl ether mol­ecules are slightly elongated and suggest a stretching of the octa­hedral geometry. This distortion could be attributed, on the one hand, to steric effects caused by the methyl substituents. On the other hand, the elongation of the Mg1—O1 bond could be explained in terms of electronic factors due to the higher electronegativity of carbon compared to hydrogen. The higher electron density in the C—O bond in comparison to the H—O bond leads to a weaker coordination of the dimethyl ether oxygen atom to the magnesium center. These elongated axial coordinations are in contrast to the structure of the magnesium(II) bromide hexa­hydrate, where all coordinations from the water mol­ecules are equal (YIKLAH; Hennings *et al.*, 2013 ▸). The directly coordinating dimethyl ether mol­ecules in **1** show longer C—O bond lengths [C1—O1 = 1.441 (5) Å, C2—O1 = 1.433 (6) Å] than those in the outer sphere [C3—O4 = 1.416 (6) Å, C4—O4 = 1.422 (6) Å]. Due to the coordination, the electron density could be shifted from the oxygen atom O1 to the O1—Mg1 coordination, causing a weakening of the C—O1 bonds. The bond lengths of the dimethyl ether mol­ecules in the outer sphere are consistent with data from the literature (Allen *et al.*, 1987 ▸).

## 3. Supra­molecular features

**Figure 2:**
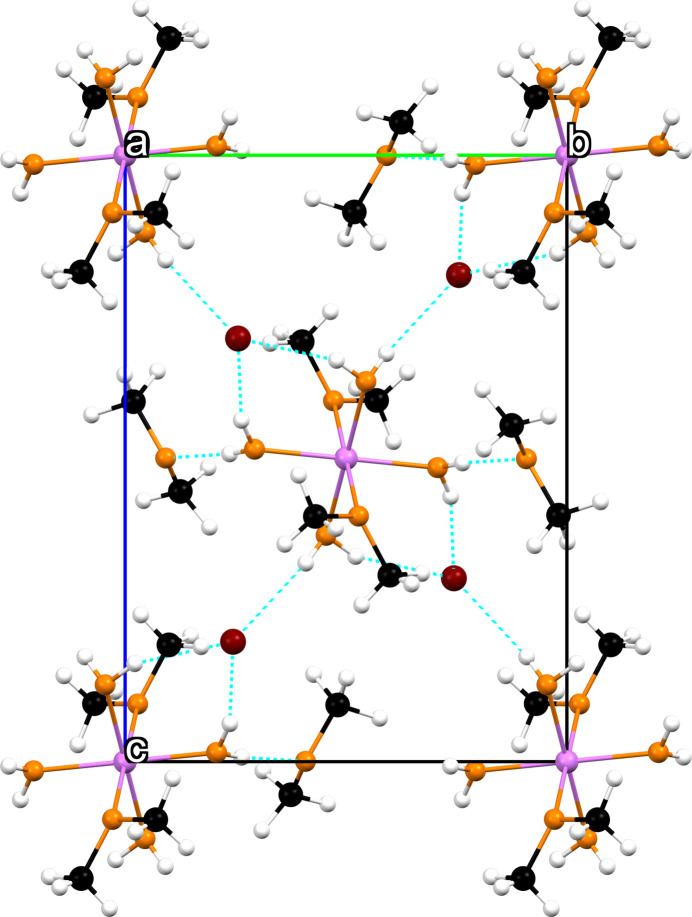
The mol­ecular packing of **1** viewed along the *a* axis with the unit cell shown as a black outline. Hydrogen bonds are shown as dashed blue lines.

The crystal packing of complex **1** is shown in Fig. 2 ▸. When observing the non-directly coordinating DME mol­ecules, a relatively short distance H3*A*⋯O4 of 1.83 (6) Å can be seen, which indicates a hydrogen bond (Table 2 ▸). Regarding the high volatility of dimethyl ether, the presence of these weakly co-coordinating mol­ecules in this aggregate is quite unusual. Similar inter­actions can be seen between H2*B*⋯Br1 [2.41 (8) Å] and H3*B*⋯Br1 [2.40 (7) Å]. The bromide anions are slightly displaced from the equatorial plane formed by the water mol­ecules, as can be seen from the angle of 85.44 (8)° for O1—Mg1⋯Br1. This could be explained by inter­molecular inter­actions, for example, hydrogen bonds.

**Figure 3:**
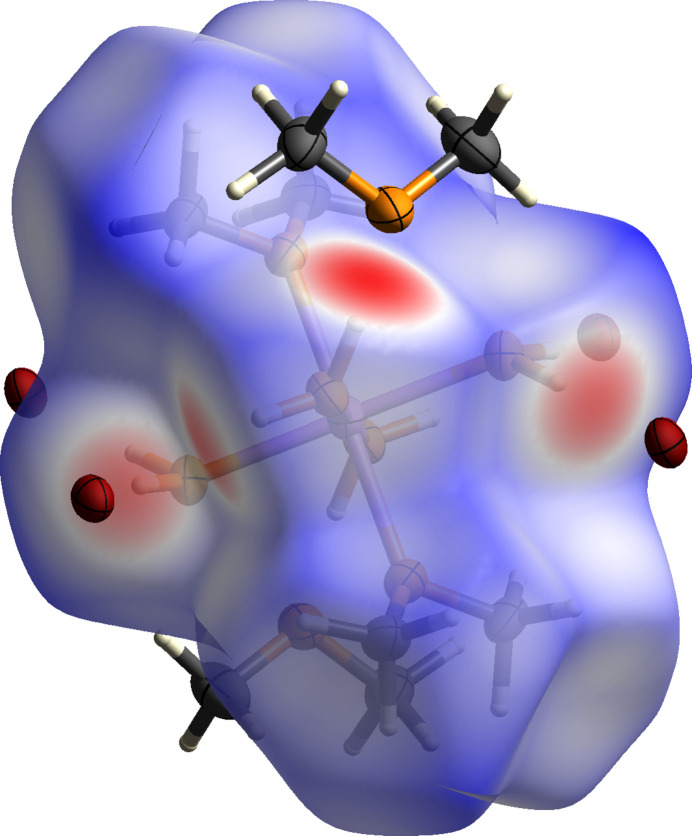
Hirshfeld surface analysis of **1** showing close contacts in the crystal.

**Figure 4:**
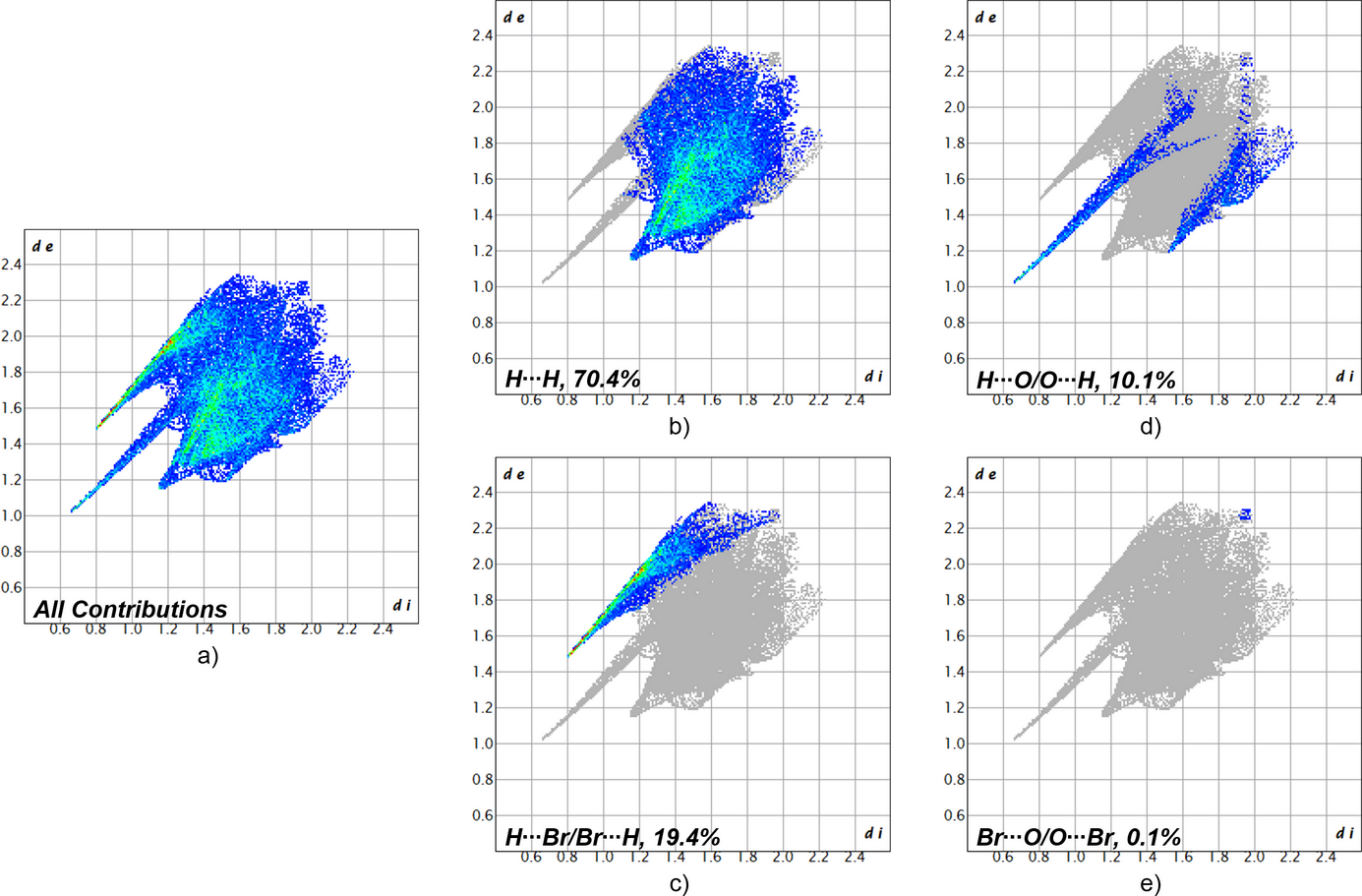
Two-dimensional fingerprint plots for compound **1**, showing (*a*) all contributions and (*b*)–(*e*) contributions between specific inter­acting atom pairs (blue areas).

To better understand the inter­molecular inter­actions and to investigate, which inter­molecular inter­action is dominating the packing of **1**, a Hirshfeld surface analysis (Spackman & Jayatilaka, 2009 ▸) was carried out. The surface and the corresponding fingerprint plots (McKinnon *et al.*, 2007 ▸) were calculated using *CrystalExplorer21* (Spackman *et al.*, 2021 ▸). Fig. 3 ▸ illustrates the Hirshfeld surface mapped over *d*_norm_ in the range from −0.71 to 1.31 arbitrary units. The red areas represent the closest contacts, which correspond to hydrogen bonds. The contributions of the respective inter­molecular inter­actions are visualized by the two-dimensional fingerprint plots shown in Fig. 4 ▸. The H⋯H inter­actions can be identified as the most significant inter­actions for the packing in the crystal structure of **1** (70.4%), followed by the H⋯Br inter­actions, contributing 19.4% and the H⋯O inter­actions with a contribution of 10.1%. The Br⋯O inter­actions, with a contribution of 0.1%, are less impactful. Based on this analysis, the H⋯H inter­actions could be identified as the most significant inter­actions of the crystal packing, whereas the hydrogen bonds represent the closest contacts between the mol­ecules.

## 4. Database survey

A search of the Cambridge Structural Database (Groom *et al.*, 2016 ▸; WebCSD February 2026) revealed several structures of magnesium(II) complexes, for example, a complex, where the magnesium ion is coordinated by two bromide anions in the axial position and four tetra­hydro­furan (THF) ligands in the equatorial position (ZZZVBQ04; Stern *et al.*, 2010 ▸). Instead of the THF ligands, another complex contains the more sterically demanding tetra­hydro­pyran (THP) ligands (OCARAO; Schüler *et al.*, 2021 ▸).

Further research reveals a more similar structure to complex **1** containing two water mol­ecules, four THF mol­ecules and two bromide anions (THFMGB; Sarma *et al.*, 1977 ▸). Another crystal structure with uncoordinated ether mol­ecules in the outer sphere consists of two different cationic magnesium moieties with two [MnCl_4_]^2–^ counter-ions. While one of the magnesium centers is coordinated by four THF mol­ecules and two water mol­ecules, the other is coordinated by two THF ligands and four water mol­ecules (NUSREY; Sobota *et al.*, 1998 ▸). The latter is a coordinated cationic domain that is very similar to the one found in complex **1**, which contains dimethyl ether ligands instead of THF. As already mentioned, a search for magnesium(II) complexes with dimethyl ether as a ligand revealed only one structure, complex **2** (KIRWAK; Kim *et al.*, 2007 ▸). In addition, two lithium halide complexes with DME ligands are known, for example (AQIKUK, AQIKOE; Hättasch *et al.*, 2025 ▸). The absence of further structures with dimethyl ether as a ligand highlights the untapped potential of investigating such compounds.

## 5. Synthesis and crystallization

To ensure safe handling of dimethyl ether in liquid form, the reaction was performed at low temperatures due to its low boiling point.

MgBr_2_ (16.0 mg, 0.090 mmol, 1.00 eq.), dissolved in THF, was used as a starting material for the synthesis of complex **1**. The solvent was removed from this reagent under reduced pressure. Dimethyl ether (1 ml) was added to the remaining salt MgBr_2_ at 223 K. After complete solvation of the salt, the reaction vessel was stored at 193 K. Compound **1** crystallized after four days in the form of colorless blocks, which were suitable for X-ray diffraction. The crystals are temperature sensitive, and were picked at 193 K. Since the complex **1** contains water, residual moisture must have been present for the compound to crystallize, although the source of water is unknown.

## 6. Refinement

Crystal data, data collection and structure refinement details are summarized in Table 3 ▸. The hydrogen atoms were located in difference maps and refined freely with isotropic displacement parameters.

## Supplementary Material

Crystal structure: contains datablock(s) I. DOI: 10.1107/S205698902600366X/hb8210sup1.cif

Structure factors: contains datablock(s) I. DOI: 10.1107/S205698902600366X/hb8210Isup2.hkl

CCDC reference: 2544504

Additional supporting information:  crystallographic information; 3D view; checkCIF report

## Figures and Tables

**Table 1:** Selected geometric parameters (Å, °)

Mg1—O1	2.140 (3)	Mg1—O2	2.060 (3)
Mg1—O3	2.026 (3)		
			
O3—Mg1—O1^i^	89.88 (12)	O3—Mg1—O2^i^	89.94 (13)
O3—Mg1—O1	90.11 (12)	O2—Mg1—O1	89.20 (12)
O3—Mg1—O2	90.06 (13)	O2^i^—Mg1—O1	90.80 (12)

Symmetry code: (i) 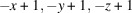.

**Table 2:** Hydrogen-bond geometry (Å, °)

*D*—H⋯*A*	*D*—H	H⋯*A*	*D*⋯*A*	*D*—H⋯*A*
O2—H2*A*⋯Br1^ii^	0.79 (6)	2.48 (5)	3.268 (3)	173 (4)
O2—H2*B*⋯Br1	0.89 (7)	2.41 (8)	3.258 (3)	159 (7)
O3—H3*A*⋯O4	0.85 (6)	1.83 (6)	2.664 (5)	170 (6)
O3—H3*B*⋯Br1	0.89 (7)	2.40 (7)	3.255 (3)	163 (5)

Symmetry code: (ii) 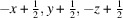.

**Table 3:** Experimental details

Crystal data	
Chemical formula	[Mg(C_2_H_6_O)_2_(H_2_O)_4_]Br_2_·2C_2_H_6_O
*M*_r_	440.46
Crystal system, space group	Monoclinic, *P*2_1_/*n*
Temperature (K)	100
*a*, *b*, *c* (Å)	8.089 (3), 9.494 (3), 13.351 (5)
β (°)	102.606 (16)
*V* (Å^3^)	1000.6 (6)
*Z*	2
Radiation type	Mo *K*α
μ (mm^−1^)	4.11
Crystal size (mm)	0.24 × 0.18 × 0.15

Data collection	
Diffractometer	Bruker D8 VENTURE area detector
Absorption correction	Multi-scan (*SADABS*; Krause *et al.*, 2015 ▸)
*T*_min_, *T*_max_	0.329, 0.491
No. of measured, independent and observed [*I* > 2σ(*I*)] reflections	18018, 2223, 1612
*R*_int_	0.060
(sin θ/λ)_max_ (Å^−1^)	0.644

Refinement	
*R*[*F*^2^ > 2σ(*F*^2^)], *wR*(*F*^2^), *S*	0.043, 0.116, 1.05
No. of reflections	2223
No. of parameters	152
H-atom treatment	All H-atom parameters refined
Δρ_max_, Δρ_min_ (e Å^−3^)	0.86, −0.88

Computer programs: *APEX6* and *SAINT* (Bruker, 2016 ▸), *SHELXT* (Sheldrick, 2015*a* ▸), *SHELXL* (Sheldrick, 2015*b* ▸) and *OLEX2* (Dolomanov *et al.*, 2009 ▸).

## supplementary crystallographic information

### Tetraaquabis(dimethyl ether)magnesium(II) dibromide dimethyl ether disolvate Crystal data

**Table:**

[Mg(C_2_H_6_O)_2_(H_2_O)_4_]Br_2_·2C_2_H_6_O	*F*(000) = 452
*M**_r_* = 440.46	*D*_x_ = 1.462 Mg m^−^^3^
Monoclinic, *P*2_1_/*n*	Mo *K*α radiation, λ = 0.71073 Å
*a* = 8.089 (3) Å	Cell parameters from 3601 reflections
*b* = 9.494 (3) Å	θ = 2.7–27.0°
*c* = 13.351 (5) Å	µ = 4.11 mm^−^^1^
β = 102.606 (16)°	*T* = 100 K
*V* = 1000.6 (6) Å^3^	Block, clear colourless
*Z* = 2	0.24 × 0.18 × 0.15 mm

### Tetraaquabis(dimethyl ether)magnesium(II) dibromide dimethyl ether disolvate Data collection

**Table:**

Bruker D8 VENTURE area detector diffractometer	2223 independent reflections
Radiation source: microfocus sealed X-ray tube, INCOATEC microfocus sealed tube, Iys 3.0	1612 reflections with *I* > 2σ(*I*)
Multilayer optics monochromator	*R*_int_ = 0.060
Detector resolution: 10.4167 pixels mm^-1^	θ_max_ = 27.2°, θ_min_ = 2.7°
ω and φ scans	*h* = −10→10
Absorption correction: multi-scan (SADABS; Krause *et al.*, 2015)	*k* = −12→12
*T*_min_ = 0.329, *T*_max_ = 0.491	*l* = −17→17
18018 measured reflections	

### Tetraaquabis(dimethyl ether)magnesium(II) dibromide dimethyl ether disolvate Refinement

**Table:**

Refinement on *F*^2^	Primary atom site location: dual
Least-squares matrix: full	Hydrogen site location: difference Fourier map
*R*[*F*^2^ > 2σ(*F*^2^)] = 0.043	All H-atom parameters refined
*wR*(*F*^2^) = 0.116	*w* = 1/[σ^2^(*F*_o_^2^) + (0.0542*P*)^2^ + 1.9217*P*] where *P* = (*F*_o_^2^ + 2*F*_c_^2^)/3
*S* = 1.05	(Δ/σ)_max_ < 0.001
2223 reflections	Δρ_max_ = 0.86 e Å^−^^3^
152 parameters	Δρ_min_ = −0.88 e Å^−^^3^
0 restraints	

### Tetraaquabis(dimethyl ether)magnesium(II) dibromide dimethyl ether disolvate Special details

**Table:**

Geometry. All esds (except the esd in the dihedral angle between two l.s. planes) are estimated using the full covariance matrix. The cell esds are taken into account individually in the estimation of esds in distances, angles and torsion angles; correlations between esds in cell parameters are only used when they are defined by crystal symmetry. An approximate (isotropic) treatment of cell esds is used for estimating esds involving l.s. planes.

### Tetraaquabis(dimethyl ether)magnesium(II) dibromide dimethyl ether disolvate Fractional atomic coordinates and isotropic or equivalent isotropic displacement parameters (Å^2^)

**Table:**

	*x*	*y*	*z*	*U*_iso_*/*U*_eq_	
Br1	0.10246 (5)	0.25568 (4)	0.30234 (3)	0.03415 (16)	
Mg1	0.500000	0.500000	0.500000	0.0284 (4)	
O1	0.3210 (4)	0.5284 (3)	0.5950 (2)	0.0341 (7)	
O3	0.4396 (4)	0.2929 (3)	0.4848 (2)	0.0326 (7)	
O2	0.3116 (4)	0.5452 (3)	0.3732 (2)	0.0323 (7)	
O4	0.6731 (4)	0.0916 (4)	0.4994 (2)	0.0452 (8)	
C1	0.3682 (6)	0.5991 (5)	0.6925 (3)	0.0362 (10)	
C2	0.1863 (6)	0.4306 (6)	0.5957 (4)	0.0416 (11)	
C4	0.6744 (7)	0.0189 (6)	0.4065 (4)	0.0462 (12)	
C3	0.8387 (7)	0.1249 (7)	0.5545 (5)	0.0547 (14)	
H3C	0.891 (6)	0.188 (6)	0.515 (4)	0.035 (13)*	
H2C	0.225 (6)	0.361 (5)	0.642 (3)	0.028 (12)*	
H1A	0.261 (5)	0.648 (5)	0.707 (3)	0.029 (11)*	
H1B	0.398 (6)	0.535 (5)	0.742 (3)	0.029 (12)*	
H4A	0.736 (7)	0.073 (6)	0.356 (4)	0.060 (17)*	
H2D	0.096 (7)	0.477 (5)	0.616 (4)	0.043 (14)*	
H1C	0.461 (6)	0.672 (5)	0.690 (4)	0.040 (13)*	
H2A	0.328 (7)	0.591 (6)	0.327 (4)	0.045 (16)*	
H3A	0.522 (8)	0.236 (6)	0.493 (5)	0.053 (17)*	
H3B	0.353 (9)	0.263 (6)	0.437 (5)	0.064 (19)*	
H2E	0.139 (6)	0.393 (5)	0.528 (4)	0.031 (11)*	
H3D	0.887 (7)	0.041 (6)	0.570 (4)	0.043 (14)*	
H4B	0.556 (7)	−0.001 (6)	0.362 (4)	0.052 (15)*	
H4C	0.740 (8)	−0.076 (7)	0.428 (5)	0.073 (19)*	
H2B	0.247 (10)	0.478 (8)	0.338 (6)	0.10 (3)*	
H3E	0.833 (9)	0.191 (8)	0.613 (5)	0.08 (2)*	

### Tetraaquabis(dimethyl ether)magnesium(II) dibromide dimethyl ether disolvate Atomic displacement parameters (Å^2^)

**Table:**

	*U*^11^	*U*^22^	*U*^33^	*U*^12^	*U*^13^	*U*^23^
Br1	0.0322 (2)	0.0351 (2)	0.0323 (2)	0.00003 (18)	0.00085 (16)	−0.00621 (18)
Mg1	0.0288 (9)	0.0306 (10)	0.0249 (9)	0.0005 (8)	0.0037 (7)	0.0004 (8)
O1	0.0320 (15)	0.0421 (17)	0.0287 (15)	−0.0023 (13)	0.0078 (12)	−0.0026 (13)
O3	0.0282 (14)	0.0334 (15)	0.0333 (16)	0.0026 (13)	0.0004 (12)	0.0007 (13)
O2	0.0363 (16)	0.0327 (16)	0.0259 (15)	−0.0007 (13)	0.0020 (12)	0.0020 (13)
O4	0.0383 (17)	0.055 (2)	0.0397 (18)	0.0101 (15)	0.0029 (14)	−0.0117 (16)
C1	0.046 (3)	0.038 (2)	0.027 (2)	0.004 (2)	0.0117 (19)	−0.0024 (19)
C2	0.034 (2)	0.053 (3)	0.039 (3)	−0.004 (2)	0.011 (2)	−0.004 (2)
C4	0.056 (3)	0.045 (3)	0.037 (3)	0.007 (2)	0.009 (2)	−0.004 (2)
C3	0.042 (3)	0.050 (3)	0.066 (4)	0.005 (3)	−0.001 (3)	−0.005 (3)

### Tetraaquabis(dimethyl ether)magnesium(II) dibromide dimethyl ether disolvate Geometric parameters (Å, º)

**Table:**

Mg1—O1	2.140 (3)	O4—C3	1.416 (6)
Mg1—O1^i^	2.140 (3)	C1—H1A	1.04 (4)
Mg1—O3^i^	2.026 (3)	C1—H1B	0.89 (5)
Mg1—O3	2.026 (3)	C1—H1C	1.02 (5)
Mg1—O2	2.060 (3)	C2—H2C	0.91 (5)
Mg1—O2^i^	2.060 (3)	C2—H2D	0.94 (5)
O1—C1	1.441 (5)	C2—H2E	0.97 (5)
O1—C2	1.433 (6)	C4—H4A	1.05 (6)
O3—H3A	0.85 (6)	C4—H4B	1.03 (5)
O3—H3B	0.88 (7)	C4—H4C	1.05 (7)
O2—H2A	0.79 (6)	C3—H3C	0.96 (5)
O2—H2B	0.89 (8)	C3—H3D	0.89 (5)
O4—C4	1.422 (6)	C3—H3E	1.01 (7)
			
O1^i^—Mg1—O1	180.0	O1—C1—H1A	108 (2)
O3—Mg1—O1^i^	89.88 (12)	O1—C1—H1B	109 (3)
O3^i^—Mg1—O1^i^	90.12 (12)	O1—C1—H1C	109 (3)
O3—Mg1—O1	90.11 (12)	H1A—C1—H1B	106 (4)
O3^i^—Mg1—O1	89.88 (12)	H1A—C1—H1C	111 (4)
O3—Mg1—O3^i^	180.0	H1B—C1—H1C	114 (4)
O3—Mg1—O2	90.06 (13)	O1—C2—H2C	109 (3)
O3—Mg1—O2^i^	89.94 (13)	O1—C2—H2D	110 (3)
O3^i^—Mg1—O2^i^	90.06 (13)	O1—C2—H2E	112 (3)
O3^i^—Mg1—O2	89.94 (13)	H2C—C2—H2D	108 (4)
O2—Mg1—O1	89.20 (12)	H2C—C2—H2E	112 (4)
O2^i^—Mg1—O1	90.80 (12)	H2D—C2—H2E	105 (4)
O2—Mg1—O1^i^	90.80 (12)	O4—C4—H4A	114 (3)
O2^i^—Mg1—O1^i^	89.20 (12)	O4—C4—H4B	114 (3)
O2—Mg1—O2^i^	180.0	O4—C4—H4C	106 (3)
C1—O1—Mg1	120.9 (3)	H4A—C4—H4B	104 (4)
C2—O1—Mg1	122.4 (3)	H4A—C4—H4C	109 (5)
C2—O1—C1	110.4 (3)	H4B—C4—H4C	111 (4)
Mg1—O3—H3A	116 (4)	O4—C3—H3C	110 (3)
Mg1—O3—H3B	121 (4)	O4—C3—H3D	104 (3)
H3A—O3—H3B	112 (5)	O4—C3—H3E	110 (4)
Mg1—O2—H2A	122 (4)	H3C—C3—H3D	117 (5)
Mg1—O2—H2B	122 (5)	H3C—C3—H3E	98 (5)
H2A—O2—H2B	100 (6)	H3D—C3—H3E	118 (5)
C3—O4—C4	112.1 (4)		

Symmetry code: (i) −*x*+1, −*y*+1, −*z*+1.

### Tetraaquabis(dimethyl ether)magnesium(II) dibromide dimethyl ether disolvate Hydrogen-bond geometry (Å, º)

**Table:**

*D*—H···*A*	*D*—H	H···*A*	*D*···*A*	*D*—H···*A*
O2—H2*A*···Br1^ii^	0.79 (6)	2.48 (5)	3.268 (3)	173 (4)
O2—H2*B*···Br1	0.89 (7)	2.41 (8)	3.258 (3)	159 (7)
O3—H3*A*···O4	0.85 (6)	1.83 (6)	2.664 (5)	170 (6)
O3—H3*B*···Br1	0.89 (7)	2.40 (7)	3.255 (3)	163 (5)

Symmetry code: (ii) −*x*+1/2, *y*+1/2, −*z*+1/2.
